# Diagnosis or prognosis? An umbrella review of mid‐trimester cervical length and spontaneous preterm birth

**DOI:** 10.1111/1471-0528.17443

**Published:** 2023-03-20

**Authors:** Kelly Hughes, Heather Ford, Shakila Thangaratinam, Shaun Brennecke, Ben W. Mol, Rui Wang

**Affiliations:** ^1^ Department of Obstetrics and Gynaecology Monash University Melbourne Victoria Australia; ^2^ WHO Collaborating Centre for Women's Health, Institute of Translational Medicine University of Birmingham Birmingham UK; ^3^ Department of Obstetrics and Gynaecology The University of Melbourne Melbourne Victoria Australia; ^4^ Department of Maternal‐Fetal Medicine & Pregnancy Research Centre Royal Women's Hospital Melbourne Victoria Australia; ^5^ Aberdeen Centre for Women's Health Research, School of Medicine, Medical Sciences and Nutrition University of Aberdeen Aberdeen UK

**Keywords:** cervical length, preterm birth, prognosis, systematic review, ultrasonography

## Abstract

**Background:**

Cervical length is widely used to assess a woman's risk of spontaneous preterm birth (SPTB).

**Objectives:**

To summarise and critically appraise the evidence from systematic reviews on the prognostic capacity of transvaginal sonographic cervical length in the second trimester in asymptomatic women with singleton or twin pregnancy.

**Search strategy:**

Searches were performed in Medline, Embase, CINAHL and grey literature from 1 January 1995 to 6 July 2021, including keywords ‘cervical length’, ‘preterm birth’, ‘obstetric labour, premature’, ‘review’ and others, without language restriction.

**Selection criteria:**

We included systematic reviews including women who did not receive treatments to reduce SPTB risk.

**Data collection and analysis:**

From 2472 articles, 14 systematic reviews were included. Summary statistics were independently extracted by two reviewers, tabulated and analysed descriptively. The ROBIS tool was used to evaluate risk of bias of included systematic reviews.

**Main results:**

Twelve reviews performed meta‐analyses: two were reported as systematic reviews of prognostic factor studies, ten used diagnostic test accuracy methodology. Ten systematic reviews were at high or unclear risk of bias. Meta‐analyses reported up to 80 combinations of cervical length, gestational age at measurement and definition of preterm birth. Cervical length was consistently associated with SPTB, with a likelihood ratio for a positive test of 1.70–142.

**Conclusions:**

The ability of cervical length to predict SPTB is a prognostic research question; systematic reviews typically analysed diagnostic test accuracy. Individual participant data meta‐analysis using prognostic factor research methods is recommended to better quantify how well transvaginal ultrasonographic cervical length can predict SPTB.

## Abstract 2

**Linked article:** This article is commented on by Andrew Combs, pp. 880 in this issue. To view this mini commentary visit https://doi.org/10.1111/1471-0528.17459.

## 1 INTRODUCTION

Preterm birth (before 37 weeks of gestation) is the leading cause of neonatal mortality worldwide, and the second‐leading cause of death in children under five.^1^ Survivors are at increased risk of a range of respiratory, sensory and neurodevelopmental disorders,^2^ obesity and cardiovascular disease.^3^ Although survival and developmental outcomes of children born preterm have improved due to advances in neonatal care, progress in the prevention of spontaneous preterm birth (SPTB) has been relatively limited.^4^

A shortened cervix in the second trimester of pregnancy has been recognised as a risk indicator for SPTB for more than 30 years,^5^ but the advent of transvaginal ultrasound provided a more reliable measure.^6^ Despite a multitude of prognostic studies, the predictive capacity of a cervical length measurement remains unclear because of varying findings among different study populations and conflicting definitions of short cervix and preterm birth.^7^, ^8^, ^9^ It is known that risk of SPTB increases as cervical length decreases,^7^ but even so, the majority of women with a short cervix will go on to deliver at term.^10^ This may explain, in part, the discrepancy in clinical guidelines between different countries and the cautiousness of their recommendations.^11^, ^12^, ^13^, ^14^, ^15^ A clinician would ideally be able to use the cervical length to help stratify a woman's risk of SPTB and to plan further surveillance or selectively offer preventive treatments (vaginal progesterone, cerclage or pessary) to reduce that risk.

The volume of literature is such that numerous systematic reviews have been published, attempting to guide antenatal care providers in the clinical application and predictive utility of transvaginal ultrasound cervical length. However, the number of review articles is also very large, with variable quality and scope, which does little to achieve the stated aim. A contemporary approach to synthesising the large amounts of information available and providing clear guidance on important topics in health care is to perform an overview of the existing systematic reviews, or umbrella review.^16^, ^17^

We conducted this umbrella review to summarise and critically appraise published systematic reviews assessing the value of transvaginal ultrasound cervical length in predicting SPTB in asymptomatic women with singleton or multiple pregnancy in the second trimester who had not received prophylactic treatment to reduce their SPTB risk. We aimed to use the outcome to suggest optimal clinical application of cervical length measurement and future directions for research.

## 2 METHODS

### 2.1 Protocol and registration

The protocol of this overview of systematic reviews was registered with PROSPERO (CRD42020138502) and the reporting is in line with the PRISMA statement.^18^

### 2.2 Patient and public involvement

Patient and public involvement was not sought as part of this review.

### 2.3 Core outcome sets

No core outcome set could be used in this review because of the scope of the research question and the analysis of literature that often pre‐dated the existence of a relevant core outcome set.^19^

### 2.4 Information sources and search strategy

The search strategy was developed in consultation with a specialist librarian and was applied without language restrictions. The search key terms included: cervix or cervical, uterine cervical incompetence, cervical length measurement, ultrasonography, preterm birth or delivery or labo(u)r, and review. Details of the search strategy are presented in Appendix S1. We searched Medline, Embase, CINAHL and LILACS databases from 1 January 1995 to 6 July 2021. In addition, we searched the Cochrane database, PROSPERO register, JBI Database of Systematic Reviews and Implementation Reports, Database of Abstracts of Reviews of Effects and Google Scholar for grey literature. We performed citation tracking on all reviews.

### 2.5 Eligibility criteria and study screening

We included systematic reviews of asymptomatic pregnant women in their second trimester with a singleton or twin pregnancy, with or without additional risk factors for SPTB, who underwent transvaginal ultrasound cervical length measurement but did not receive preventive treatments. Systematic reviews evaluating the prognostic value of transvaginal ultrasound cervical length, either alone or as part of a wider research question, were eligible. Systematic reviews were defined as those with explicit intent ‘to identify appraise and synthesize all the empirical evidence that meets pre‐specified eligibility criteria to answer a specific research question’.^20^ We searched beyond 1995 with no language restrictions applied. We excluded systematic reviews presented as conference abstracts only, clinical practice guidelines and narrative reviews. We excluded systematic reviews that were unable to report on the presence of symptoms of preterm labour, and those that were unable to report on whether preterm births were spontaneous or iatrogenic. We excluded systematic reviews where cervical length was measured by transabdominal, translabial or transperineal routes because of the lack of reliability of these methods.^21^, ^22^, ^23^ We also excluded systematic reviews where the cervical length measurement resulted in the use of treatments to reduce the risk of SPTB. Grey literature was eligible for inclusion if meeting the criteria for a systematic review and if complete text was available.

Studies were screened by title and abstract by two reviewers (KH, RW). Initial screening aimed to identify reviews of any kind that examined the predictive utility of transvaginal ultrasound cervical length in asymptomatic pregnant women in the second trimester. Full‐text review was performed by two investigators (KH, HF) independently. Disagreements were resolved by consultation with a third reviewer (BWM or RW), or by consensus.

### 2.6 Data extraction

Data were extracted independently by two reviewers (KH, HF), using a form based on the Johanna Briggs Institute data extraction form^24^ (Appendix S2). The data items include number of participants, type of population (inclusion/exclusion criteria), details on the exposure (cervical length measurement, including gestational age at measurement and definition of short cervix [in mm]), details on the outcome (definition of SPTB [in weeks] and summary statistics on the outcomes) and methods for data synthesis. Cervical length measurements during the first and third trimester are beyond the scope of this review and therefore these data were not extracted.

### 2.7 Risk of bias assessment

ROBIS (Risk Of Bias In Systematic reviews)^25^ was used as the primary tool for risk of bias assessment and was performed independently by two reviewers (KH, HF). ROBIS assesses the following domains: study eligibility, identification and selection of studies, data collection and study appraisal, synthesis and findings. AMSTAR‐2 (A MeaSurement Tool to Assess systematic Reviews‐2) was also used as a supplementary tool, assessing for use of ideal research methods in systematic reviews that include non‐randomised studies, including research question components, use of a prospectively prepared research protocol, literature search strategy, study selection and data extraction in duplicate, reporting of funding sources and several more. The ‘overall confidence rating’ derived from AMSTAR‐2^26^ was applied to each review.

### 2.8 Data synthesis

The key characteristics of systematic reviews, including design, participants, prognostic factor of interest (gestational age at measurement of cervical length, cervical length cutoffs), outcomes, timing of prediction, sample size and effect measures were summarised and tabulated descriptively. Summary statistics of different systematic reviews were tabulated and visualised, noting that the unit of analysis was a systematic review instead of a primary study and therefore data from primary studies were not re‐extracted for synthesis. Results across different systematic reviews that measured the same populations and used matching cutoffs for gestational age at measurement, short cervical length and definition of SPTB were also summarised.

### 2.9 Dealing with overlapping studies

Given the aim was to provide an overview of all the available systematic reviews on this topic, we decided to include all relevant systematic reviews including overlapping primary studies.^27^ We mapped the included studies in different systematic reviews in a league table (Appendix S3).

## 3 RESULTS

### 3.1 Study selection

**FIGURE 1:**
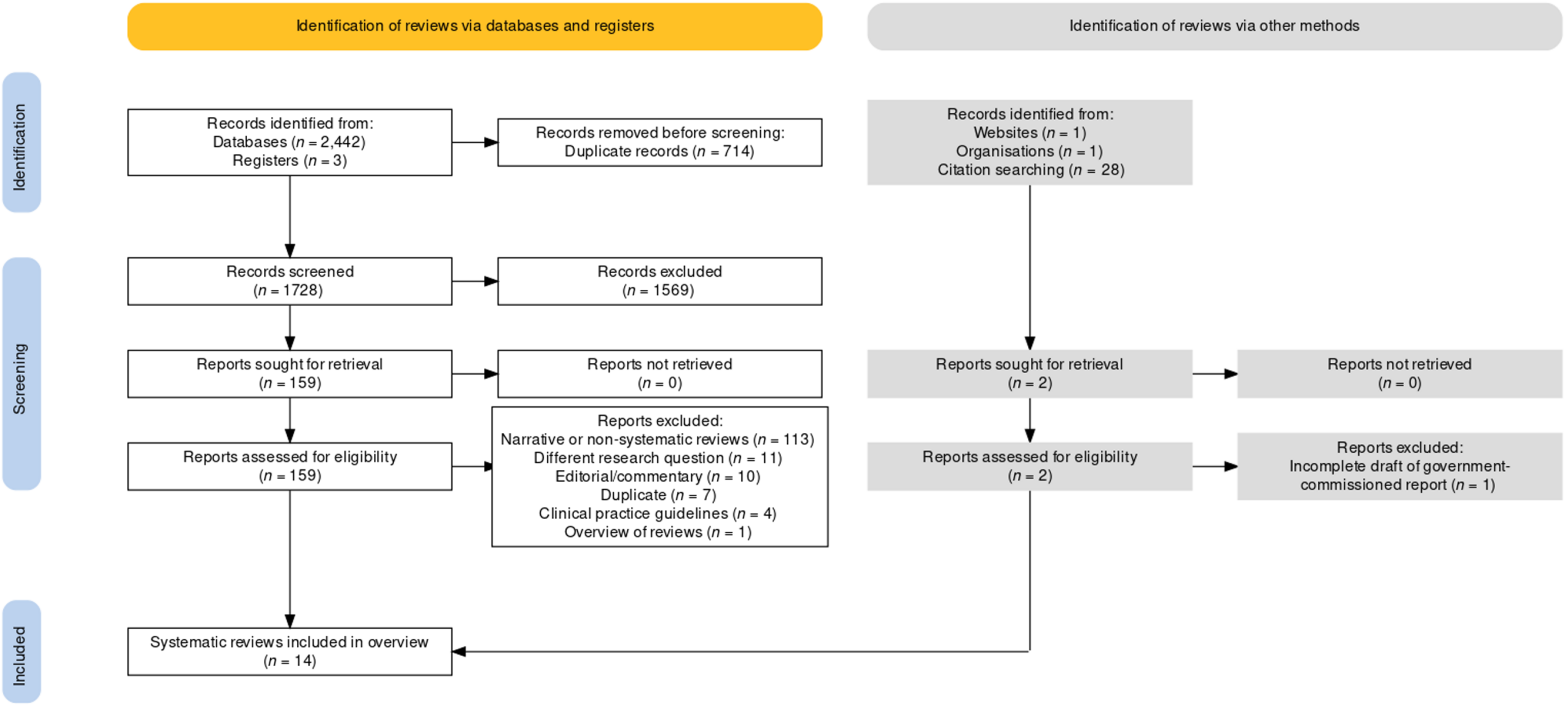
PRISMA 2020 flow chart.

The PRISMA flow diagram is presented in Figure 1.^28^ The search yielded 2475 items in total, of which 1569 were excluded after removal of duplicates and screening titles and abstracts. The remaining 161 full‐text reviews were assessed for eligibility. One hundred and forty‐seven were excluded for the following reasons: 113 were narrative reviews, 11 had a different research question, ten were editorials or commentaries only, four were clinical practice guidelines, one was an incomplete draft of a government‐commissioned review, and one performed a qualitative overview of reviews assessing both cervical length and fetal fibronectin and, because of its earlier publication date, only contained two relevant systematic reviews (also in our search results) and did not contribute any additional data. A list of the excluded reviews is available in Appendix S2.

### 3.2 Characteristics of included systematic reviews

Table 1 summarises the key characteristics of the 14 systematic reviews,^29^, ^30^, ^31^, ^32^, ^33^, ^34^, ^35^, ^36^, ^37^, ^38^, ^39^, ^40^, ^41^, ^42^ including design, participants, prognostic factor of interest (gestational ages at measurement of cervical length, cervical length cutoffs, outcomes), timing of prediction, sample size and effect measures for meta‐analysis. Two systematic reviews did not include meta‐analysis.^39^, ^40^ Of the 12 systematic reviews with meta‐analysis, two^38^, ^41^ were based on individual participant data, with cervical length as a prognostic factor; the other ten were based on aggregate data, considering cervical length as a diagnostic accuracy test. Eight assessed asymptomatic women only and six addressed both symptomatic and asymptomatic women; separate analysis of patient groups within these papers allowed us to consider only the data relevant to our research question. Five included singleton studies only; four included twin studies only, and five reported on both singleton and twin pregnancies. Thirteen systematic reviews assessed primary studies that used a single transvaginal measurement of cervical length and the other evaluated the change in cervical length over time.^35^

**TABLE 1:** Summary table of systematic reviews with meta‐analysis.

Systematic review	Population	No. of studies	No. of participants	Index prognostic factor (cervical length)		Outcome definition (spontaneous preterm birth, < gestational age in weeks)	Timing (GA at measurement)	Methods		
				Cervical length cutoff values (mm)	No. of studies per cutoff set			Design	Data source	Data synthesis
Leitich 1999	Singleton, normal risk^a^	6	106–3694	20, 25, 29, 30, 34, 35, 39, 40, 45, 50, 55	1–2	35, 37	20, means: 20^+4^, 23^+4^, 27^+6^; 29	Diagnostic	Aggregate	Study sensitivities/ specificities plotted as ROC curve
	Twin, unselected	1	253	25	1	32, 35, 37	23, 27.5 (mean)			
Honest 2003	Singleton, normal or high‐risk^a^	18	50–6877	10, 15, 20, 22, 27, 29, 30, 35, 39, 40, 45, 50, 60	1–5	32, 34, 37	<20, 20–24, >24	Diagnostic	Aggregate	sROC curves, LR+/LR−
	Twin, unselected or high‐risk	11	43–434	15, 20, 25, 30, 35, 40, 45	1–4	32, 34, 37	<20, 20–24, >24			
Krupa 2006	Singleton and twin	6	32–2915	Not described	Not described	Not described	Not described	n/a	n/a	n/a
Crane 2008	Singleton, high‐risk	14	42–1476	10, 15, 20, 25, 30, 35, 40, 45	1–9	26, 28, 30, 32, 34, 35, 37	<20, 12–24, 14–18, 14–24, 15–20, 15–24, 16–19, 18–22, 20–24, <24, 24–28, 24–30, 25–29, <32	Diagnostic	Aggregate	Fixed effects meta‐analysis, summary LR+/LR−
Honest 2009	Singleton, normal or high‐risk^a^	13	69–6334	15, 20, 22, 25, 27, 30, 35, 39	1–2	34, 37	<20, 20–24, >24	Diagnostic	Aggregate	Meta‐analysis technique not further described. Summary LR+/LR−
Conde‐Agudelo 2010	Twin, unselected (one study post‐selective reduction of triplets)^a^	16	18–1135	15–20, 15.9 ± 0.3, 18, 18–24, 18–26, 18–36, 20, 20–25, 20–32, 21–23, 22–24, 23, 23–33, 24, 24–26, 24–36, 25–28, 25–32, 26–28, 28, 32, <34, 38	2–6	34, 37	20–24	Diagnostic	Aggregate	Bivariate random‐effects meta‐analysis. sROC curves, sensitivity, specificity, LR+/LR−
Domin 2010	Singleton, normal or high‐risk	23	57–6877	20, 25, 33.15	ns	35, 37	<20, >20, 14–24	Diagnostic	Aggregate	Random‐effects meta‐analysis, ROC curves. AUC, sensitivity, specificity, LR+/−
Lim 2011	Twin, with or without additional risk factors	16	14–434	20, 25, 30, 35	not reported	<30, 30–34, <34	<20, 20–24, >24	Diagnostic	Aggregate	Bivariate regression analysis. Sensitivity, specificity
Honest 2012 (data from 2009)	Singleton, normal or high‐risk^a^	ns	ns	15, 20, 22, 25, 30, 32.5	1–5	34, 37	14–20, 20–24	Diagnostic	Aggregate	Meta‐analysis technique not described. Summary LR+/−
Kleinrouweler 2013	Singleton, previous SPTB^a^	7	37−76 476	15, 15–20, 20–25, 25–30, ≥30	ns	32, 34, 37	<15, 15–20, 20–25, 25–30, ≥30	Prognostic	Individual participant	Univariable analysis, wo‐level random intercept logistic regression models, Cox regression analysis, % risk
Conde‐Agudelo 2014	Twin, unselected (one study post‐selective reduction of triplets)	16 (single measure)	434–1955	20, 25	3–5	28, 32, 34, 37	20–24	Diagnostic	Aggregate	Meta‐analysis technique not described. ROC curve or sensitivity, specificity, LR+
		7 (shortening over time)	1004	Any shortening	ns	28–36	ns			
Barros Silva 2014	Singleton (normal or high‐risk) or twin	21	80–5068	10, 15, 20, 22, 23.3, 25, 26, 28, 29, 30, 35	1–8	26, 28, 30, 32, 33, 34, 35, 37	18, 18–21, 18–22, 18–24, 20–24, 21–24, 22–24, 23, 24	n/a	n/a	n/a
Conde‐Agudelo 2015	Singleton, normal or high‐risk	6	68–2531	Any shortening	1–5	35, 37	Disregarded	Diagnostic	Aggregate	Bivariate random effects meta‐analysis, sROC curves. Sensitivity, specificity, LR+
	Twin, unselected	7	20–209	Any shortening	3–7	28, 30, 32, 34				
Kindinger 2016	Twin, without additional risk factors^a^	12	22–1138	5, 10, 15, 20, 25, 30, 35, 40	12	28, 28–32, 32–36, ≥36	18–26	Prognostic	Individual participant	Multinomial logistic regression, multinomial log‐linear model. % risk

*Note*: Where two lines of data are listed for a single review, the first line indicates the thresholds reported by the review. The second line indicates the thresholds reported in the included primary studies.

Abbreviations: AUC, area under the curve; GA, gestational age; LR, likelihood ratio; n/a, not applicable; ns, not specified; ROC, receiver operating characteristic; sROC, summary receiver operating characteristic.

^a^ Includes unpublished data.

Systematic reviews included between 6 and 23 primary studies, reporting data on 1312–26 474 participants. The ten aggregate data meta‐analyses performed multiple analyses, reporting from 3 to 80 combinations of cutoffs (gestational age at measurement, cervical length and gestational age at delivery), which summarised data from between one and nine studies (75 and 6047 participants) per combination, as outlined in Table 2.

**TABLE 2:** Reported summary statistics.

Author	Year	Plurality	GA at measurement (weeks)	Cutoff CL (mm)	Definition of PTB (weeks)	AUC	Sensitivity	Specificity	LR+	LR−	% Probability of PTB	Notes
Honest	2003	Singleton	<20	25	32				4.10	0.75		
Crane	2008	Singleton	<20	25	32				3.18			
Honest	2003	Singleton	<20	15	34				30.53	0.90		Same single study result
Crane	2008	Singleton	<20	15	35				30.53			Same single study result
Honest	2012	Singleton	14–20	15	34				142.86	0.89		
Honest	2003	Singleton	<20	20	34				14.47	0.90		
Domin	2010	Singleton	14–25	≤20	35	0.89	22.1	98.2	12.4	0.40		
Honest	2003	Singleton	<20	25	34				6.29	0.79		
Honest	2009/2012	Singleton	14–20	25	34				13.38	0.80		
Crane	2008	Singleton	<20	25	35				4.31	0.68		
Domin	2010	Singleton	14–25	≤25	35	0.85	33.3	95.9	6.30	0.65		
Honest	2003	Singleton	<20	30	34				2.84	0.75		
Honest	2012	Singleton	14–20	30	34				2.48	0.81		
Crane	2008	Singleton	<20	30	35				3.23			
Leitich	1999	Singleton	20	30	37		33	91				
Honest	2003	Singleton	<20	30	37				3.77	0.73		
Honest	2003	Singleton	20–24	20	34				7.64	0.79		
Honest	2012	Singleton	20–24	20	34				7.64	0.79		
Domin	2010	Singleton	14–25	≤20	35	0.89	22.1	98.2	12.4	0.40		
Honest	2003	Singleton	20–24	25	32				4.19	0.40		
Crane	2008	Singleton	20–24	25	32				2.38			
Honest	2003	Singleton	20–24	25	34				4.40	0.67		
Honest	2009	Singleton	20–24	25	34				4.68	0.68		
Crane	2008	Singleton	20–24	25	35				2.78	0.55		
Domin	2010	Singleton	14–25	≤25	35	0.85	33.3	95.9	6.30	0.65		
Honest	2003	Singleton	20–24	30	34				2.28	0.60		
Crane	2008	Singleton	20–24	30	35				1.75			
Honest	2009/2012	Singleton	20–24	32.5	37				3.99	0.33		
Domin	2010	Singleton	14–25	≤33.15	37	0.83	32.7	90	4.90	0.60		
Honest	2003	Singleton	>24	15	34				7.08	0.93		
Crane	2008	Singleton	>24	15	35				5.64			
Honest	2003	Singleton	>24	20	34				5.83	0.81		
Crane	2008	Singleton	>24	20	35				5.16			
Honest	2003	Singleton	>24	25	34				4.07	0.62		
Crane	2008	Singleton	>24	25	35				4.01	0.72		
Honest	2003	Singleton	>24	30	34				2.70	0.52		
Crane	2008	Singleton	>24	30	35				2.23			
Honest	2003	Singleton	>24	30	37				2.49	0.79		
Crane	2008	Singleton	>24	30	37				2.27			
Honest	2003	Twin	20	20	34				59.89	0.71		
Lim	2011	Twin	not specified	20	34		30	94	*5.00*	*0.74*		
Conde‐Agudelo	2014	Twin	20–24	20	34		29	97	9.00	0.74		
Kindinger	2016	Twin	20	20	32–36						20.60	
Kindinger	2016	Twin	22	20	32–36						26.50	
Kindinger	2016	Twin	24	20	32–36						33.10	
Conde‐Agudelo	2010	Twin	20–24	≤25	32	0.8	54	91	6.00	0.51		
Conde‐Agudelo	2014	Twin	20–24	25	32				10.10	0.64		
Kindinger	2016	Twin	20	25	28–32						35.00	
Kindinger	2016	Twin	22	25	28–32						32.40	
Kindinger	2016	Twin	24	25	28–32						29.30	
Honest	2003	Twin	20–24	25	34				5.02	0.75		
Lim	2011	Twin	not specified	25	34		36	94	*6.00*	*0.68*		
Conde‐Agudelo	2014	Twin	20–24	≤25	34		40	93	5.80	0.65		
Kindinger	2016	Twin	20	25	32–36						25.00	
Kindinger	2016	Twin	22	25	32–36						31.10	
Kindinger	2016	Twin	24	25	32–36						37.60	
Honest	2003	Twin	20–24	30	34				2.31	0.69		
Conde‐Agudelo	2010	Twin	20–24	30	34		56	81	3.00	0.55		
Kindinger	2016	Twin	20	30	32–36						28.50	
Kindinger	2016	Twin	22	30	32–36						34.30	
Kindinger	2016	Twin	24	30	32–36						40.30	
Honest	2003	Twin	>24	25	34				1.82	0.83		
Conde‐Agudelo	2010	Twin	>24	25	34		44	81	2.30	0.70		
Kindinger	2016	Twin	24	25	32–36						37.60	
Kindinger	2016	Twin	26	25	32–36						44.30	
Honest	2003	Twin	>24	25	37				1.89	0.73		
Conde‐Agudelo	2010	Twin	>24	25	37		43	77	1.80	0.75		
Conde‐Agudelo	2014	Twin	20–24	25	37		21	95	4.40	0.80		
Kindinger	2016	Twin	24	25	≥36						19.00	
Kindinger	2016	Twin	26	25	≥36						19.40	
Honest	2003	Twin	>24	30	34				2.11	0.61		
Lim	2011	Twin	not specified	30	34		41	87	*3.15*	*0.32*		
Kindinger	2016	Twin	24	30	32–36						40.30	
Kindinger	2016	Twin	26	30	32–36						46.30	
Honest	2003	Twin	>24	35	34				1.84	0.29		
Lim	2011	Twin	not specified	35	34		78	66	*2.29*	*0.33*		
Kindinger	2016	Twin	24	35	32–36						40.70	
Kindinger	2016	Twin	26	35	32–36						45.90	

*Note*: Cells are blank where a summary statistic was not reported. Italicised numbers were not reported in the original paper, but calculated from data in the review.

Abbreviations: AUC, area under the curve; CL, cervical length; GA, gestational age; LR, likelihood ratio; PTB, preterm birth.

Cervical length was measured between 12 and 30 (or more) weeks of gestation. This wide variation in gestational age at measurement was most commonly addressed by reporting summary statistics for a gestational age range; however, one group calculated mean gestational ages at measurement.^32^ Up to 22 different gestational ages (or age ranges) at cervical length measurement were reported in the primary studies included in a single review.^35^

A variety of cutoffs (ranging from 5 to 60 mm) were used for defining a short cervix, with 20 mm (*n* = 9), 25 mm (*n* = 10) and 30 mm (*n* = 7) the most used. Up to 23 different cutoffs were reported in the primary studies included in a single systematic review (Table 1).^36^

Definitions of spontaneous preterm birth (the primary outcome) also varied among the included studies, with up to seven thresholds reported per review.^33^ The most common cutoffs were less than 34 and less than 37 weeks of gestation.

Of the few studies using the same statistical analysis methods that also reported similar cutoffs for cervical length and SPTB, Lim et al.^37^ and Conde‐Agudelo et al.^42^ reported comparable results, as did Lim et al.^37^ and Conde‐Agudelo et al.^36^ (Table 2), however gestational age at measurement was not specified in the paper by Lim et al. because of limitations of the methodology. The similar findings may be explained by the proportion of overlapping studies, shown in Appendix S4; two groups re‐reported their own data in later publications.^30^, ^31^, ^35^, ^42^

Due to heterogeneous reporting in the primary studies, between 2 and 13 studies were excluded from meta‐analyses of aggregate data. For the two individual participant data meta‐analyses, 11 of 23 and 7 of possibly 247 (number not clearly specified) eligible studies were included due to inability or unwillingness to share data.

Among the ten systematic reviews of diagnostic test accuracy, the most reported statistics were summary likelihood ratios (*n* = 7), summary receiver operating characteristic (ROC) curves (*n* = 7) and summary sensitivities and specificities (*n* = 5). Three reviews performed bivariate meta‐analysis. One plotted each study's reported sensitivity and specificity in the style of an ROC curve, without generating a summary ROC curve.^43^

### 3.3 Risk of bias assessments

**FIGURE 2:**
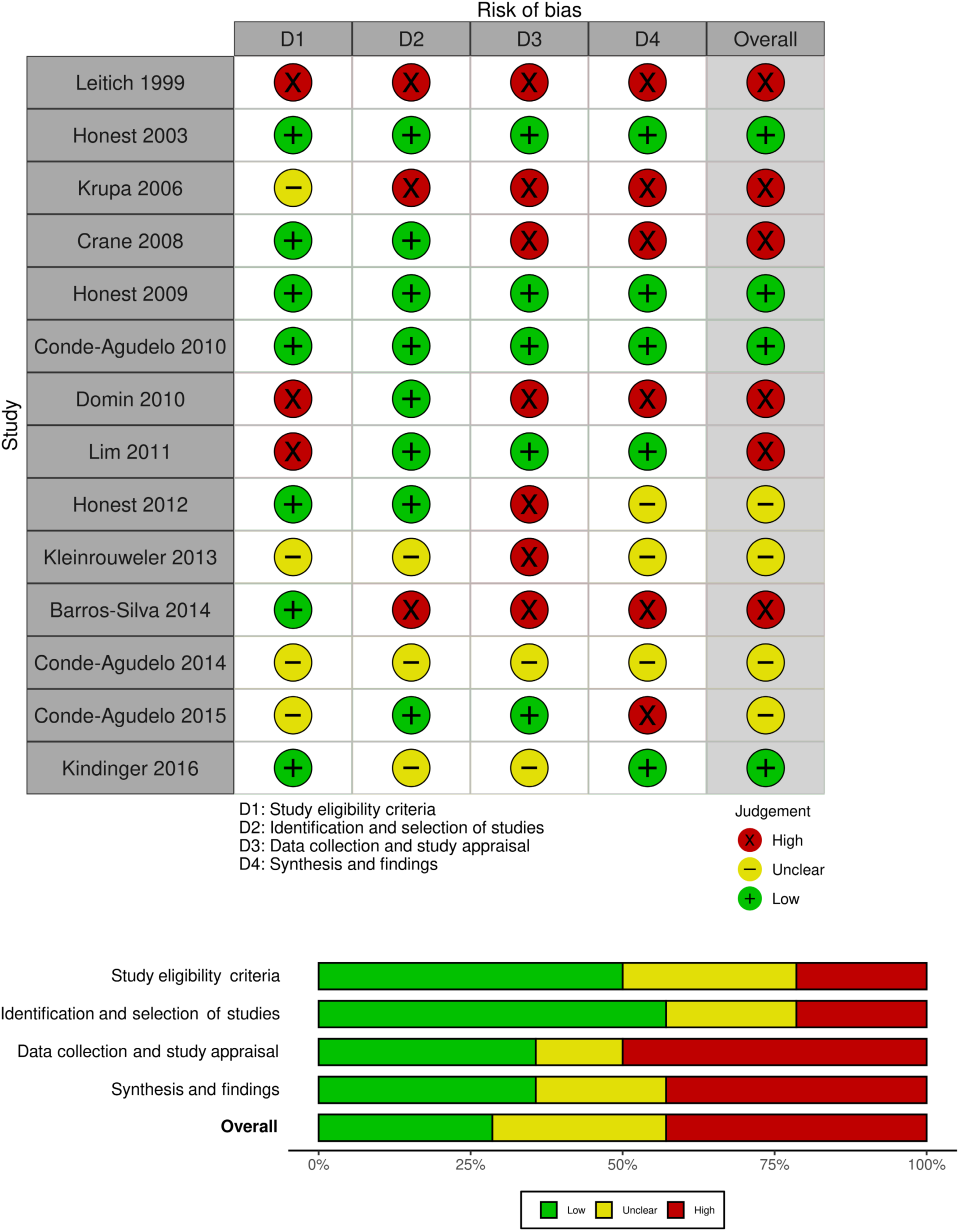
ROBIS traffic light and summary plot.

Results from risk of bias assessment are shown in Figure 2. Only four of 14 reviews were assessed as having a low overall risk of bias overall with ROBIS, six reviews were rated at high risk of bias and four had an unclear risk of bias. Eight of 14 systematic reviews performed well in ROBIS domains of identification and selection of studies, and seven in study eligibility criteria. AMSTAR‐2 results are available in Appendix S5.

### 3.4 Single cervical length measurement and preterm birth in singleton pregnancies

Based on four systematic reviews of women, the likelihood ratio of a positive test (LR+) for cervical length of 25 mm or less before 20 weeks of gestation (except for Domin et al. at 14–25 weeks) for preterm birth before 34 or 35 weeks were 4.31–13.38 and the likelihood ratio of a negative test (LR−) was 0.65–0.80. For preterm birth before 32 weeks of gestation, the LR+ from two systematic reviews was 3.18–4.10 and the LR− was 0.75. Additional common combinations of thresholds are shown in Table 2 and Appendix S2.

### 3.5 Single cervical length measurement and preterm birth in twin pregnancies

Three systematic reviews showed that for cervical length of 25 mm or less measured at 20–24 weeks of gestation predicting preterm birth before 34 weeks, the LR+ was 5.02–6.00 and the LR− 0.65–0.75, sensitivity 36–40% and specificity 93–94%. Details of additional results are summarised in Table 2 and Appendix S2.

### 3.6 Cervical length change and preterm birth

Conde‐Agudelo et al.'s most recent review^35^ examined change in cervical length over time as a diagnostic test, reporting on 13 different combinations of variables. The extent of cervical shortening was substituted for cervical length: ‘any shortening’ over the study period, shortening to a threshold, or a percentage shortening. Gestational age at measurement encompassed wide ranges (10–28 weeks at initial measurement through to 20–30 weeks at final measurement).

The group described, for women with twin pregnancy, 47% sensitivity, 88% specificity and LR+ 4.00 of any shortening of cervical length for predicting SPTB before 34 weeks. Findings were similar for 20–25% shortening in a similar population (47% sensitivity, 87% specificity and LR+ 3.80). An earlier review by the same authors listed a range of findings for any cervical shortening (15–75% sensitivity, 70–90% specificity, LR+ 1.60–5.50, LR− 0.30–0.80) predicting SPTB between less than 28 and 36 weeks.^42^

## 4 DISCUSSION

### 4.1 Main findings

Cervical length was consistently associated with SPTB, but the LR+ was between 1.70 and 142 depending on the cutoffs used. Using the second‐trimester transvaginal ultrasound to predict SPTB is a prognostic research question as opposed to a diagnostic question, as SPTB is a future outcome, not detection of a condition present at the time of measurement. However, of the 14 included systematic reviews, over 85% reported the research question as a diagnostic accuracy test instead of a prognostic question, and over 70% had a high or unclear risk of bias. Included meta‐analyses reported up to 80 combinations of cutoffs of cervical length, gestational age at measurement and definition of preterm birth. Consequently, transvaginal ultrasound showed variable degrees of association with SPTB.

### 4.2 Clinical and research implications

We have identified several issues in the current literature that could be improved in the future. First, most systematic reviews considered the research question as a diagnostic, instead of a prognostic question. Therefore, confounding could not be accounted for in the analysis and the reported predictive value of cervical length might reflect the influence of other factors instead of cervical length itself. Guidance on prognosis research, including the PROGRESS framework,^44^, ^45^, ^46^, ^47^ should be followed in future studies. Second, the preponderance of narrative reviews among those published in the past two decades, an issue likewise observed in other areas of medicine.^48^ Although the limitations of narrative reviews are well‐acknowledged,^48^, ^49^ they are frequently the basis of recommendations for clinical practice. Third, overall risk of bias in the included systematic reviews was high or unclear in the majority, and also in many assessment domains, perhaps due to word count restrictions and insufficient reporting in primary studies.^44^, ^45^, ^46^, ^47^ Lastly, we observed up to 80 combinations of cutoffs of cervical length, gestational age at measurement and definition of preterm birth in included meta‐analyses. Dichotomisation of continuous variables results in a loss of data^50^ and makes comparison of findings across studies difficult. Statistical analysis plans are best made in conjunction with biostatisticians, and cervical length should be ideally treated as a continuous variable in analyses.

Recommended prognosis research methodology includes reporting of prognostic effect measures (hazard or odds ratios) instead of diagnostic effect measures (sensitivity and specificity), and adjusting for other potential prognostic factors.^51^ In addition, as mentioned above, clinicians are urged to avoid dichotomising variables for simplicity or convenience due to the loss of data that ensues.^50^ The importance of gaining additional days of gestation, especially in extreme prematurity for example, is not adequately reflected by simply dichotomising data into ‘preterm birth less than 37 weeks of gestation’ or ‘term birth’. We propose to treat the outcome SPTB as a time‐to‐event outcome instead of a binary outcome so that SPTB at different gestational ages can be differentiated in the analysis.

A single prognostic factor is often insufficient to accurately determine a person's risk;^45^ most reviews appreciated this in their findings. Prognostic models, if carefully developed, calibrated and externally validated, may be more useful in practice.^45^ However, to date, multiple‐marker prediction models have not proved overly successful in predicting SPTB,^52^, ^53^ and are therefore not widely used in clinical practice, leaving the clinician with few evidence‐based options for risk assessment.

### 4.3 Strengths and limitations

This overview of systematic reviews is underpinned by a broad, well‐designed literature search, and adheres to PRISMA guidelines. We offer novel insights into the limitations of study design and statistical methods previously used in this literature. A potential limitation in this overview was that title/abstract screening was performed by only one reviewer (however, a low threshold was used to proceed to full‐text review), although it was unlikely that eligible systematic reviews were missed given our comprehensive search strategy and citation tracking. Significant overlap between included primary studies was observed (some authors used the same set of studies across two reviews),^30^, ^31^, ^36^, ^42^ and although this is acknowledged in our results, there is no agreed method for dealing with this issue.

### 4.4 Interpretation

The literature assessed in this review reports a broad spectrum of possible outcomes in women with a short cervix. The likelihood ratios may be interpreted as indicating a woman with a ‘short’ cervix is between 1.70 and 142 times more likely to develop the condition (SPTB) as a woman with a ‘long’ cervix, depending on the thresholds used. However, these are very imprecise figures that cannot be directly applied in clinical practice. Furthermore, this assumes that a diagnostic measure may be repurposed as a prognostic indicator. Now that prognostic factor research methods have been more completely described, we recommend quantifying risk with these tools.^44^, ^45^, ^46^, ^47^

This can be applied to other areas of research in perinatal medicine, such as the evaluation of preventive treatments to reduce SPTB risk. Hypothetically, a treatment that prolongs gestational age from 32 to 34 weeks will be discarded if the outcome is a binary outcome defined as SPTB before 37 weeks of gestation, but this 2‐week period will be captured when the outcome is considered as a time‐to‐event outcome.

Given that many studies have already been conducted in women with different risk profiles, rather than abandoning these and simply calling for more high‐quality studies, we would advocate for using this existing work by performing individual participant data meta‐analysis using prognostic research methods and considering SPTB as a time‐to‐event outcome. This approach is the optimal method of data synthesis and has the potential to overcome the important issues identified with the meta‐analyses of aggregate data (inadequate reporting, data loss, statistical methods). Additionally, it avoids the ethical quandary of failing to offer prophylactic treatment to women with a short cervix in the context of a randomised controlled trial. An issue already encountered by the authors of the individual participant data meta‐analyses, however, is an inability or unwillingness to share data, which reflects the urgent need for collaboration to improve patient outcomes and minimise research waste.^54^

## 5 CONCLUSION

Our review of the literature on transvaginal cervical length ultrasonography to predict SPTB revealed several issues, and we contend that, despite the quantity of research that has been conducted in this area, the question of how well mid‐trimester TV cervical length predicts SPTB is yet to be completely answered.

The bulk of published literature comprises narrative reviews with lower methodological rigour. The systematic reviews, nonetheless, carried significant risk of bias and reported on literature that was heterogeneous, with varying thresholds for a number of different variables. Statistical analysis in the primary studies and systematic reviews was performed to assess diagnostic test accuracy; however, cervical length is a prognostic factor that requires a different approach. Our review revealed an overall trend toward recommending transvaginal ultrasound cervical length measurement for asymptomatic women with singleton or twin pregnancy in the second trimester to predict SPTB, but most systematic reviews acknowledged that cervical length has limited ability to effectively identify many women who will go on to deliver prematurely. Likewise, most women with a short cervix will ultimately birth at term.^55^ At present, cervical length will most likely continue to be used to guide treatment decisions until it can be replaced by more precise prognostic factors or models. Individual patient data meta‐analysis has excellent potential to overcome the limitations in the existing literature, and we recommend this as the next step, using prognostic factor research methodology and analysing continuous variables.

## AUTHOR CONTRIBUTIONS

BWM, ST, SB and RW were involved in conception and supervision of this project, and approval of the article. HF assessed articles for inclusion and risk of bias. RW also assisted with development of the manuscript. KH produced the main concepts of the project, developed and conducted the literature search, acted as the first reviewer and wrote the manuscript.

## FUNDING INFORMATION

This work is funded by an Australian National Health and Medical Research Council project grant (GNT1146590). The funder was not involved in conducting research or writing the paper.

## CONFLICT OF INTEREST

BWM is supported by an NHMRC Practitioner Fellowship (GNT1082548) and reports a consultancy for ObsEva, Merck Merck KGaA, and Guerbet. Completed disclosure of interests form available to view online as supporting information.

## ETHICS APPROVAL

As no participant recruitment or new data collection was involved in this review, ethics approval was not required.

## Supporting information


Appendix S1

Appendix S2

Appendix S3

Appendix S4

Appendix S5

Data S1

Data S2

Data S3

Data S4

Data S5

Data S6

## Data Availability

Data sharing is not applicable to this article as no new data were created or analysed in this study.
