# Supplementary material for: Diagnosis or prognosis? An umbrella review of mid‐trimester cervical length and spontaneous preterm birth
Source: BJOG. 2023 Mar 20;130(8):866–79. doi: 10.1111/1471-0528.17443 (PMC10953024; doi:10.1111/1471-0528.17443)
Supplement: Supplementary file 1 — Appendix S1 [file BJO-130-866-s009.docx]

Search strategy

| **Term #** | **Search term** |
| --- | --- |
| 1 | “screening to prevent spontaneous preterm birth”.mp |
| 2 | “length of the cervix and the risk of spontaneous”.mp |
| 3 | “predictive accuracy of serial transvaginal cervical lengths”.mp |
| 4 | ((cervix or cervical) adj3 length).mp |
| 5 | Cervix Uteri/ |
| 6 | Uterine Cervical Incompetence/ |
| 7 | Cervical Length Measurement/ |
| 8 | Ultrasonography, Prenatal/ |
| 9 | 4 or 5 or 6 or 7 or 8 |
| 10 | ((pre-term or preterm or premature) adj (delivery or birth or labour or labor).mp |
| 11 | Exp Obstetric Labor, Premature/ |
| 12 | abortion, spontaneous/ |
| 13 | 10 or 11 or 12 |
| 14 | 9 and 13 |
| 15 | 4 or 7 or 8 |
| 16 | 13 and 15 |
| 17 | (review* or systematic or meta* or umbrella).mp |
| 18 | 16 and 17 |
| 19 | 14 not 16 |
| 20 | 1 and 16 |
