# Supplementary material for: Diagnosis or prognosis? An umbrella review of mid‐trimester cervical length and spontaneous preterm birth
Source: BJOG. 2023 Mar 20;130(8):866–79. doi: 10.1111/1471-0528.17443 (PMC10953024; doi:10.1111/1471-0528.17443)
Supplement: Supplementary file 3 — Appendix S3 [file BJO-130-866-s005.docx]

| **Trial** | Andersen 1990 | Tongsong 1995 | Hasegawa 1996(a) | Hasegawa 1996(b) | Iams 1996 | Berghella 1997 | Goldenberg 1998 | Heath 1998 | Taipale 1998 | Berghella 1999 | Arinami 1999 | Bouchar 1999 | Hartmann 1999 | Watson 1999 | Andrews 2000 | Cook 2000 | Hassan 2000 | Hibbard 2000 | Guzman 2001 | Iams 2001 | | Owen 2001 | To 2002 | Berghella 2003 | De Carvalho 2003 | Fukami 2003 | Berghella 2004 | Owen 2004 | Yazici 2004 | Airoldi 2005 | De Carvalho 2005 | Durnwald 2005 | Leung 2005 | Arabin 2006 | Crane 2006 | Dilek 2006 | Grgic 2006 | Hebbar 2006 | Matijevic 2006 | Ozdemir 2006 | Pires 2006 | Berghella 2007 | Bittar 2007 | Bagga 2009 | Barber 2011 | Bolt 2011 | Crane 2011 |
| --- | --- | --- | --- | --- | --- | --- | --- | --- | --- | --- | --- | --- | --- | --- | --- | --- | --- | --- | --- | --- | --- | --- | --- | --- | --- | --- | --- | --- | --- | --- | --- | --- | --- | --- | --- | --- | --- | --- | --- | --- | --- | --- | --- | --- | --- | --- | --- |
| **Systematic Review** |  |  |  |  |  |  |  |  |  |  |  |  |  |  |  |  |  |  |  |  |  |  |  |  |  |  |  |  |  |  |  |  |  |  |  |  |  |  |  |  |  |  |  |  |  |  |  |
| Leitich 1999 |  |  |  |  |  |  |  |  |  |  |  |  |  |  |  |  |  |  |  |  |  | |  |  |  |  |  |  |  |  |  |  |  |  |  |  |  |  |  |  |  |  |  |  |  |  |  |
| Honest 2003 |  |  |  |  |  |  |  |  |  |  |  |  |  |  |  |  |  |  |  |  |  | |  |  |  |  |  |  |  |  |  |  |  |  |  |  |  |  |  |  |  |  |  |  |  |  |  |
| Krupa 2006 |  |  |  |  |  |  |  |  |  |  |  |  |  |  |  |  |  |  |  |  |  | |  |  |  |  |  |  |  |  |  |  |  |  |  |  |  |  |  |  |  |  |  |  |  |  |  |
| Crane 2008 |  |  |  |  |  |  |  |  |  |  |  |  |  |  |  |  |  |  |  |  |  | |  |  |  |  |  |  |  |  |  |  |  |  |  |  |  |  |  |  |  |  |  |  |  |  |  |
| Honest 2009 |  |  |  |  |  |  |  |  |  |  |  |  |  |  |  |  |  |  |  |  |  | |  |  |  |  |  |  |  |  |  |  |  |  |  |  |  |  |  |  |  |  |  |  |  |  |  |
| Domin 2010 |  |  |  |  |  |  |  |  |  |  |  |  |  |  |  |  |  |  |  |  |  | |  |  |  |  |  |  |  |  |  |  |  |  |  |  |  |  |  |  |  |  |  |  |  |  |  |
| Honest 2012 |  |  |  |  |  |  |  |  |  |  |  |  |  |  |  |  |  |  |  |  |  | |  |  |  |  |  |  |  |  |  |  |  |  |  |  |  |  |  |  |  |  |  |  |  |  |  |
| Kleinrouweler 2013* |  |  |  |  |  |  |  |  |  |  |  |  |  |  |  |  |  |  |  |  |  | |  |  |  |  |  |  |  |  |  |  |  |  |  |  |  |  |  |  |  |  |  |  |  |  |  |
| Barros-Silva 2014 |  |  |  |  |  |  |  |  |  |  |  |  |  |  |  |  |  |  |  |  |  | |  |  |  |  |  |  |  |  |  |  |  |  |  |  |  |  |  |  |  |  |  |  |  |  |  |
| Conde-Agudelo 2015* |  |  |  |  |  |  |  |  |  |  |  |  |  |  |  |  |  |  |  |  |  | |  |  |  |  |  |  |  |  |  |  |  |  |  |  |  |  |  |  |  |  |  |  |  |  |  |

Singleton

Kleinrouweler includes unpublished data

Conde-Agudelo includes additional studies specific to change in cervical length over time

Twin

Conde-Agudelo 2015 includes additional studies specific to change in cervical length over time

Kindinger 2016 includes additional data from RCT

| **Trial** | Goldenberg 1996 | Imseis 1997 | Wennerholm 1997 | Althuisius 1998 | Grisaru- Granovsky 1998 | Souka 1999 | Guzman 2000 | Naba 2000 | Weisz 2000 | Yang 2000 | Skentou 2001 | McMahon 2002 | Soriano 2002 | Vaysierre 2002 | Gibson 2004 | Fait 2005 | Robyr 2005 | Sayin 2005 | Sperling 2005 | Arabin 2006 | To 2006 | Klein 2008 | Aboulghar 2009 | Fox 2009 | Fox 2010 | Hofmeister 2010 | Oh 2012 | Khalil 2013 | Sauvanaud 2013 | Leveque 2015 |
| --- | --- | --- | --- | --- | --- | --- | --- | --- | --- | --- | --- | --- | --- | --- | --- | --- | --- | --- | --- | --- | --- | --- | --- | --- | --- | --- | --- | --- | --- | --- |
| **Systematic Review** |  |  |  |  |  |  |  |  |  |  |  |  |  |  |  |  |  |  |  |  |  |  |  |  |  |  |  |  |  |  |
| Leitich 1999 |  |  |  |  |  |  |  |  |  |  |  |  |  |  |  |  |  |  |  |  |  |  |  |  |  |  |  |  |  |  |
| Honest 2003 |  |  |  |  |  |  |  |  |  |  |  |  |  |  |  |  |  |  |  |  |  |  |  |  |  |  |  |  |  |  |
| Conde-Agudelo 2010 |  |  |  |  |  |  |  |  |  |  |  |  |  |  |  |  |  |  |  |  |  |  |  |  |  |  |  |  |  |  |
| Lim 2011 |  |  |  |  |  |  |  |  |  |  |  |  |  |  |  |  |  |  |  |  |  |  |  |  |  |  |  |  |  |  |
| Barros-Silva 2014 |  |  |  |  |  |  |  |  |  |  |  |  |  |  |  |  |  |  |  |  |  |  |  |  |  |  |  |  |  |  |
| Conde-Agudelo 2014 |  |  |  |  |  |  |  |  |  |  |  |  |  |  |  |  |  |  |  |  |  |  |  |  |  |  |  |  |  |  |
| Conde-Agudelo 2015* |  |  |  |  |  |  |  |  |  |  |  |  |  |  |  |  |  |  |  |  |  |  |  |  |  |  |  |  |  |  |
| Kindinger 2016* |  |  |  |  |  |  |  |  |  |  |  |  |  |  |  |  |  |  |  |  |  |  |  |  |  |  |  |  |  |  |
