# Supplementary figures and images for: Diagnosis or prognosis? An umbrella review of mid‐trimester cervical length and spontaneous preterm birth
Source: BJOG. 2023 Mar 20;130(8):866–79. doi: 10.1111/1471-0528.17443 (PMC10953024; doi:10.1111/1471-0528.17443)

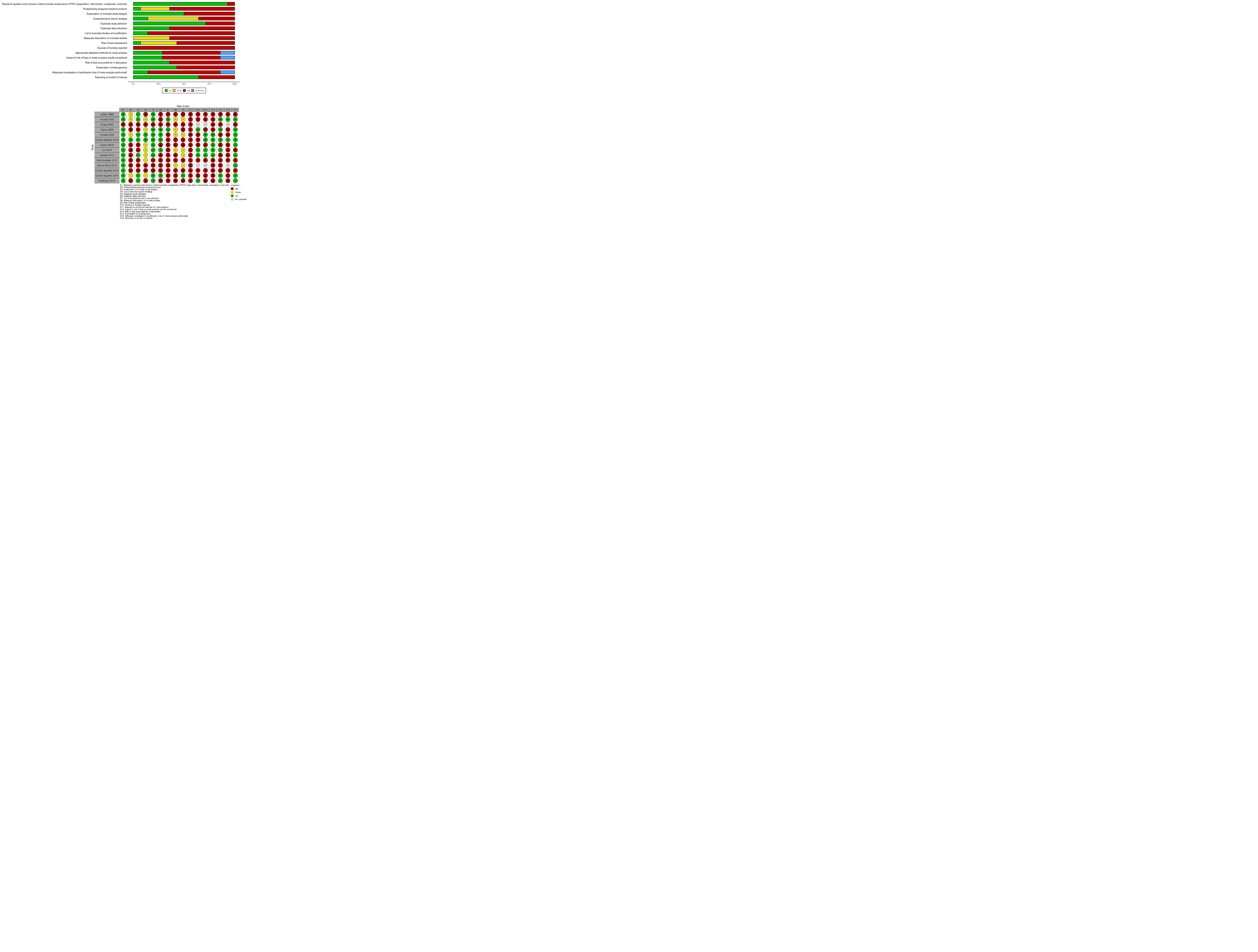

Supplement: Supplementary file 5 — Appendix S5 [file BJO-130-866-s007.png]
